# Effect of the fixed combination of valerian, lemon balm, passionflower, and butterbur extracts (Ze 185) on the prescription pattern of benzodiazepines in hospitalized psychiatric patients—A retrospective case‐control investigation

**DOI:** 10.1002/ptr.6618

**Published:** 2020-01-27

**Authors:** Martin E. Keck, Simon Nicolussi, Kerstin Spura, Cordula Blohm, Catherine Zahner, Jürgen Drewe

**Affiliations:** ^1^ Clienia Private Clinic Schlössli Oetwil Switzerland; ^2^ Psychotherapeutic Neurology Schmieder Clinic Gailingen Germany; ^3^ Medical Department Max Zeller Söhne AG Romanshorn Switzerland

**Keywords:** anxiety, benzodiazepines, depression, phyto‐anxiolytic, psychiatric disorders, Ze 185

## Abstract

Stress is an increasing problem that can result in various psychiatric and somatoform symptoms. Among others, benzodiazepines and valerian preparations are used to treat stress symptoms. The aim of this study was to investigate whether the prescription of a fixed herbal extract combination of valerian, lemon balm, passionflower, and butterbur (Ze 185) changes the prescription pattern of benzodiazepines in hospitalized psychiatric patients. In a retrospective case‐control study, anonymized medical record data from 3,252 psychiatric in‐house patients were analysed over a 3.5‐year period. Cases (*n* = 1,548) with a prescription of Ze 185 and controls (*n* = 1,704) were matched by age, gender, hospitalization interval, and main International Classification of Diseases, Version 10 F‐diagnoses. The primary objective was to investigate the effect of Ze 185 on the prescription pattern of benzodiazepines. Secondary objectives investigated the prescriptions of concomitant drugs and effectiveness of the hospital stay. Distribution of drug classes was analysed using the WHO's anatomic‐therapeutic‐chemical code. Data showed that both treatment modalities had a comparable clinical effectiveness but with significantly less prescriptions of benzodiazepines in the Ze 185 group (*p* = .006). This is of clinical importance because suitable alternatives to benzodiazepines are desirable. To obtain more support for this hypothesis, a dedicated randomized, controlled clinical trial monitoring drug safety is required.

**Abbreviations**
AMDP: Association for Methodology and Documentation in Psychiatry
ATC: Anatomic‐Therapeutic‐Chemical (WHO classification system)
CGI: Clinical Global Impression
CIS: Clinical Information System
GAF: Global Assessment of Functioning
ICD‐10: International Classification of Diseases, Version 10
RCT: Randomized Controlled Trial

## 1 INTRODUCTION

In modern societies, stress is an increasing problem that can result in various somatoform symptoms such as sleep disorders. In a Swiss survey about sleeping habits, sleep quality and the use of sedatives, 2.8% of the respondents reported that they take medication to improve sleep. The most frequently taken sedative drugs were benzodiazepines as well as valerian preparations or benzodiazepine‐like drugs (Tinguely, Landolt, & Cajochen, 2014). Long‐term use of benzodiazepines can pose important adverse effects, which should be considered. These adverse effects include drug dependence, abuse, hangover effects, cognitive and memory impairment, drowsiness, ataxia, motor incoordination, and falls (Gerlach, Maust, Leong, Mavandadi, & Oslin, 2018; Johnson & Streltzer, 2013; Woods, Katz, & Winger, 1992). Especially in elderly, a more than 50% increased risk of hip fractures was found, not to mention consequential costs (Finkle et al., 2011; Johnson & Streltzer, 2013). As a consequence of important identified safety risks, an association between benzodiazepines and an increased risk of mortality was discussed (Agarwal & Landon, 2019; Donnelly, Bracchi, Hewitt, Routledge, & Carter, 2017; Palmaro, Dupouy, & Lapeyre‐Mestre, 2015; Patorno, Glynn, Levin, Lee, & Huybrechts, 2017; Woods et al., 1992). Chronic use of benzodiazepines can lead to tolerance to the pharmacological effects and withdrawal symptoms after discontinuation of the drug. They act not only as sedatives but also have myorelaxant and anxiolytic effects. In addition, recreational use is a relevant complication (Lalive, Rudolph, Luscher, & Tan, 2011).

As stress causes various symptoms, such as anxiety, agitation, nervous tension, and sleep disorders, a single drug might be not the solution for the problem. A suitable way to treat stress‐related symptoms may be the administration of a multicomponent mixture. Herbal medicinal products contain various active ingredients by nature. In the European Union, preparations of *Valeriana officinalis* L. (Valerianaceae), *Melissa officinalis* L. (Lamiaceae), and *Passiflora incarnata* L. (Passifloraceae) are traditionally used for the treatment of nervous tension, the relief of mild symptoms of mental stress, and to aid sleep; either as single extracts or in combination with other herbal extracts (EMA/HMPC, 2013, 2014, 2016). In Switzerland, complementary medicine, including treatment methods with evidence‐based herbal medicine, is well accepted by patients and currently covered by the mandatory basic health insurance, when performed by a certified physician (Klein, Torchetti, Frei‐Erb, & Wolf, 2015). There is one authorized herbal medicinal combination product (Ze 185) that, in addition to the above‐mentioned herbal extracts, contains a dry extract of *Petasites hybridus* (L.) Gaertn., B. Mey. & Scherb (Asteraceae). *P. hybridus* is best known for its antispasmodic properties and anti‐inflammatory effect (Anon., 2001). The efficacy and safety of Ze 185 for the treatment of somatoform disorders has been confirmed in a double‐blind, randomized, placebo‐controlled clinical study (Melzer, Schrader, Brattström, Schellenberg, & Saller, 2009). In addition, an open, randomized pilot study of the effect of Ze 185 in comparison with oxazepam was undertaken in patients with psychosomatic and psychovegetative disorders (Schellenberg, Sauer, & Brattström, 2004). Further, in a double‐blind, randomized controlled clinical trial, the reaction to exam anxiety in healthy subjects was studied (Steiner & Opwis, 2000). Recently, the anxiolytic properties of Ze 185 were confirmed in a psychosocial stress paradigm: the Trier social stress test (Meier et al., 2018). These clinical studies show that Ze 185 is a well‐tolerated herbal medicinal product in the respective indications.

The aim of the current retrospective case‐control study was to investigate the effect of Ze 185 on the prescription pattern of benzodiazepines in a population of hospitalized patients with psychiatric and somatoform disorders. Special focus was given to concomitant medications to investigate whether Ze 185 could substitute synthetic drugs.

## 2 MATERIAL AND METHODS

### 2.1 Study design and setting

In this single centre retrospective case‐control study, the electronic medical records of anonymized adult in‐patients (age >18 years) were analysed, who were treated at the Clienia private clinic Schlössli in Oetwil am See, Zurich, Switzerland. Patients were hospitalized between January 2, 2010 and May 15, 2013. The clinic is specialized in psychiatric, psychotherapeutic, and psychosomatic treatments. Besides standard therapy, patients are treated with complementary medicine, such as phytotherapy, acupuncture, biofeedback, and hypnosis.

### 2.2 Ethical approval

According to Swiss law, at the time of the investigation, an ethical approval by the responsible local ethics committee of the Canton of Zurich was not necessary for the retrospective analysis of anonymized patient data (KEK, 2010).

### 2.3 Drug of interest

Ze 185 (Relaxane®) was prescribed as film‐coated tablets, which contain the fixed combination of four dry extracts: 90 mg of a 90% (w/w) ethanolic extract of *P. hybridus* (L.) Gaertn., B. Mey. & Scherb roots (DER 7–14:1); 90 mg of a 45% (*w/w*) methanolic extract of *V. officinalis* L. roots (DER 4–6:1); 90 mg of a 50% (w/w) ethanolic extract of *P. incarnata* L. herb (DER 3–6:1); 60 mg of a 20% (w/w) ethanolic extract of *M. officinalis* L. leaves (DER 2.5–3.9:1; Fingerprint analysis see Figure 3). The fixed herbal drug combination is manufactured by Zeller Medical AG (CH‐8590 Romanshorn, Switzerland) and has been registered and marketed in Switzerland since 1970 for the treatment of nervousness, nervous tension, agitation, and exam anxiety. These ailments can, amongst others, lead to the following symptoms: spasmodic gastrointestinal complaints, increased irritability, occasional trouble falling asleep, and sleeping through the night. The recommended dose is one film‐coated tablet three times daily.

### 2.4 Patient inclusion

Patients from all wards of the psychiatric clinic were included in the study. Therefore, cases and controls were selected for analysis by automatic identification from medical records using the Clinical Information System (CIS). Complete anonymization of the patient data was guaranteed for the analysis.

Cases: All patients were included in the analysis, if they were treated with any dose of Ze 185 on at least 1 day during their inpatient visit between January 2, 2010 and May 15, 2013. Controls: The control population without Ze 185 treatment was defined based on matching age (±5 years), gender, hospitalisation during the same interval (±6 months), and main International Classification of Diseases, Version 10 (ICD‐10) F‐diagnosis at the end of the clinical visit.

### 2.5 Study variables

The primary objective of the retrospective data analysis was to investigate the effect of Ze 185 on the prescription pattern of benzodiazepines in hospitalized patients with psychiatric disorders. The prescription pattern was investigated stratified by various primary F‐diagnoses according to ICD‐10 (WHO, 1992) at the end of the hospital stay.

The secondary objectives were to investigate (a) the effect of Ze 185 on the prescription pattern of other concomitantly prescribed drugs and (b) the effectiveness of the hospital stay in comparison with patients without Ze 185 treatment. This effectiveness was evaluated based on the Clinical Global Impression (CGI) score (Guy, 1976), the Global Assessment of Functioning (GAF) score (Hall, 1995), and selective symptoms of the “Association for Methodology and Documentation in Psychiatry*”* (AMDP) system (AMDP, 1982; AMDP‐CIPS, 1990). In the CGI score, higher values correspond to a condition with stronger severity and lower values to less severity. In the GAF score, lower value counts correspond to a less severe disease and higher values to a stronger severity. In the AMDP system, 140 items are counted to provide a psychopathological diagnosis. The single items of the AMDP system were scored on a 4‐point scale (0 = *not at all*, 1 = *mild*, 2 = *moderate*, and 3 = *severe*). For this retrospective data analysis, 11 items of the AMDP system were selected for detailed analysis. Four items from the category mental diagnosis (item 65 “anxious*”*; item 69 “restless”; item 82 “agitated”; item 83 “motor restlessness”) and seven items from the category somatic diagnosis (item 101 “initial insomnia”; item 102 “middle insomnia”; item 106 “decreased appetite”; item 119 “palpitations”; item 122 “sweating”; item 126 “diffuse pressure in head”; item 127 “back pain”) were chosen for further analysis.

The distribution of the drug classes was analysed using the WHO's anatomic‐therapeutic‐chemical classification system code with focus on medication with psychotropic activity.

As for safety, no data were reported in the CIS. Therefore, safety and tolerability of the patients' medication could not be evaluated.

### 2.6 Database structure

For the descriptive data analysis, medical records from the CIS were extracted to an Excel file. The information scientist of the clinic extracted and anonymized all patient data. For the anonymization, the patient information number was used by the information scientist to create a pseudo patient information number. Thus, a complete anonymization of the patient data was guaranteed for the data analyst.

### 2.7 Statistical analysis

Descriptive statistical analysis and all statistical tests used were performed using IBM SPSS Statistics for Windows, Version 21.0 (IBM Corp., Armonk, New York). The *t* test was used for normally distributed variables; otherwise, the Wilcoxon rank sum test was used. For the comparison of categorical variables, the Chi‐squared test was used. The default summary statistics for quantitative and ordinal variables were the number of observations (*n*), mean, standard deviation (*SD*), or median, as appropriate. For qualitative variables, the number and frequency of subjects with non‐missing data (*n*, %) per category were presented.

### 2.8 Study performance

This retrospective analysis was performed according to the checklist for observational studies (Strengthening the Reporting of Observational Studies in Epidemiology, STROBE; von Elm et al., 2007).

## 3 RESULTS

**Figure 1:**
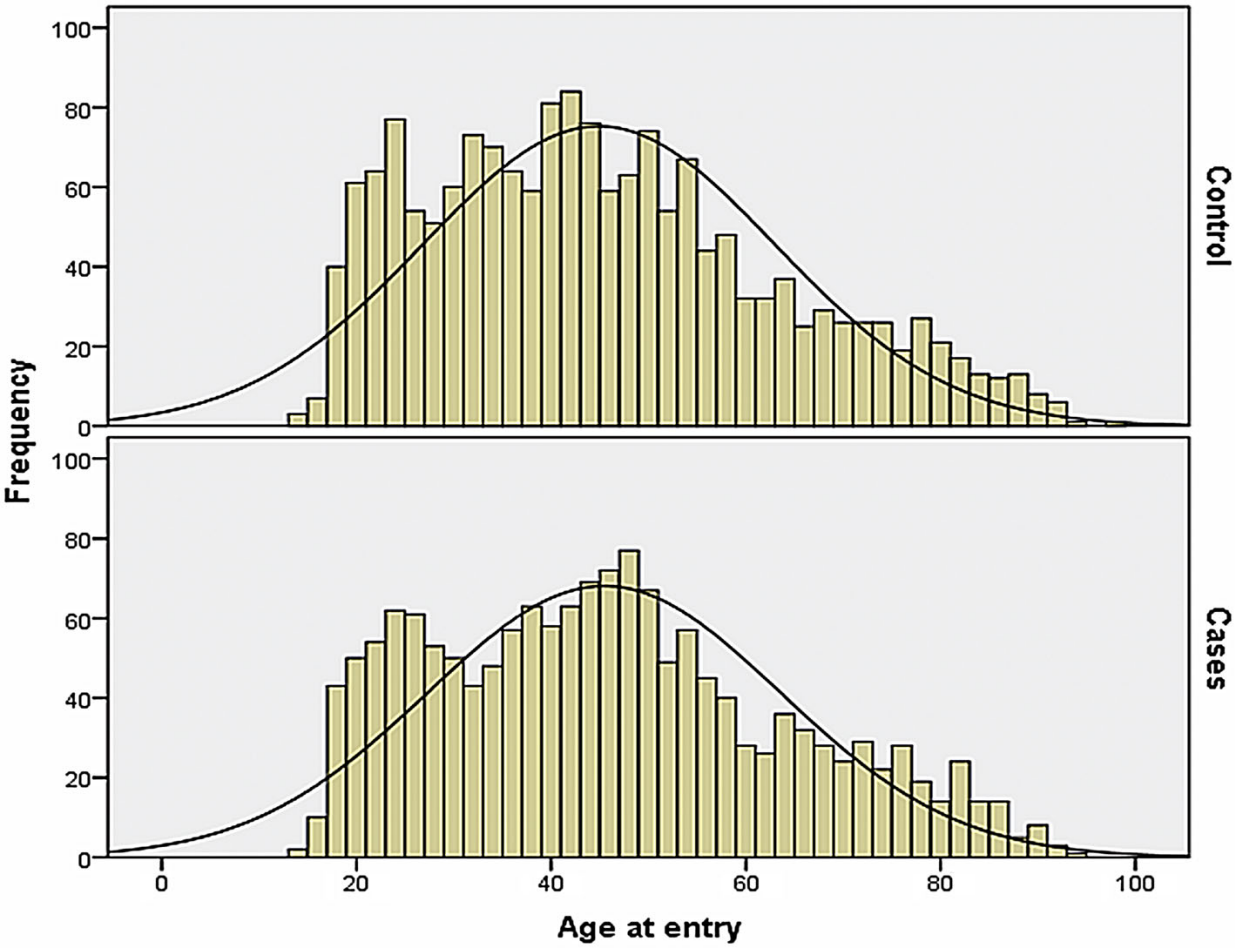
Demographic distribution of patient's age at entry. Slight bimodal distribution of age could be observed (normal distribution is given for reference) [Colour figure can be viewed at wileyonlinelibrary.com]

A total of 3,252 patients were included in the retrospective data analysis. Of these, 1,548 cases were treated with Ze 185, and 1,704 matched controls received regular medical treatment without Ze 185. Demographic characteristics are described in Table 1. A distribution of the patients' ages at entry is shown in Figure 1. In both groups, age and gender were equally distributed. The duration of the hospital stay was on average (mean ± *SD*) numerically comparable (*n* = 1,548 cases: 38.9 ± 29.6, range 1 to 205 days and *n* = 1,704 controls: 33.6 ± 29.4 days, range 1 to 185 days) but significantly different between the groups (*p* < .001).

**Table 1:** Demographic characteristics

Parameter	Cases^a^ (*n* = 1,548)	Controls^b^ (*n* = 1,704)	Total (*n* = 3,252)	*p*‐value
Age (years), mean ± *SD* (median)	45.4 ± 18.1 (44.0)	44.6 ± 18.1 (43.0)	45.2 ± 18.1 (43.0)	.390
Sex (male/female)	585/963	694/1010	1279/1973	.087
Duration of hospital stay (days), mean ± *SD* (median)	38.9 ± 29.6 (33.0)	33.6 ± 29.4 (26.0)	36.1 ± 29.6 (29.0)	<.001

a Receiving at least one prescription of Ze 185.

b Receiving no prescription of Ze 185, matched to cases.

### 3.1 F‐diagnoses

**Figure 2:**
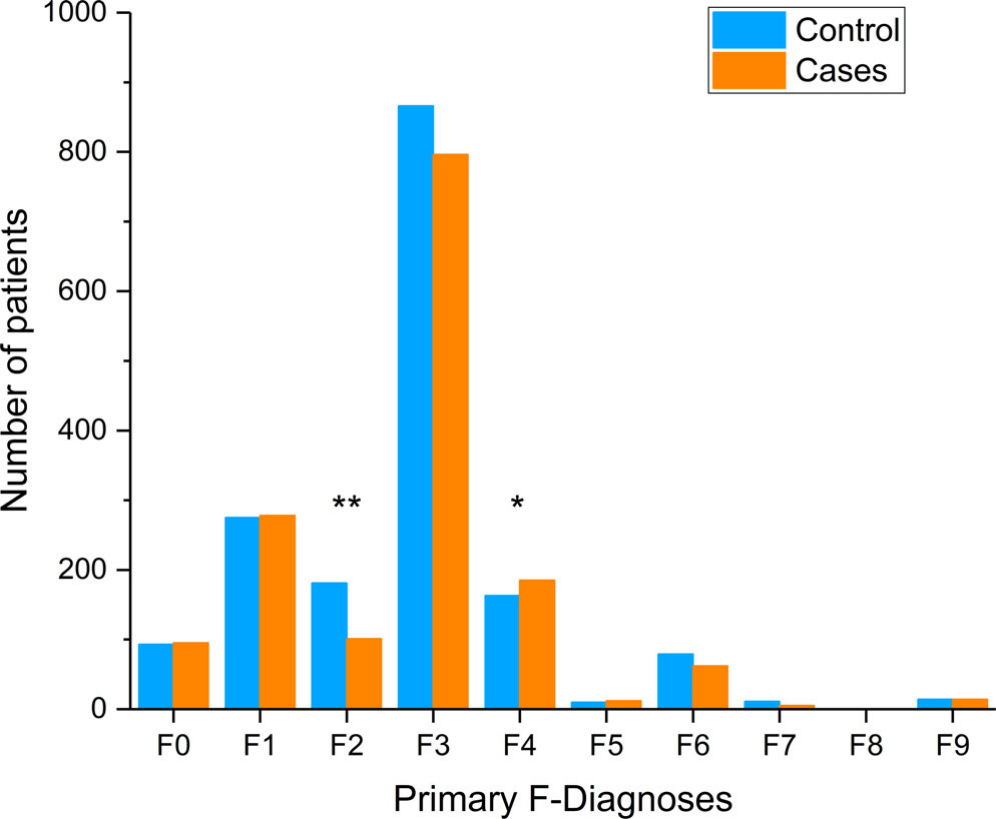
Distribution of primary F‐diagnoses according to International Classification of Diseases, Version 10. Number of patients within a group at the end of the hospital stay. Cases, *n* = 1,548: receiving at least one prescription of Ze 185, controls, *n* = 1,704: receiving no prescription of Ze 185, matched to cases; F0: Organic, including symptomatic, mental disorders; F1: Mental and behavioral disorders due to psychoactive substance use; F2: Schizophrenia, schizotypal, and delusional disorders; F3: Mood (affective) disorders; F4: Neurotic, stress‐related and somatoform disorders; F5: Behavioral syndromes associated with physiological disturbances and physical factors; F6: Disorders of adult personality and behavior; F7: Mental retardation; F8: Disorders of psychological development; F9: Behavioral and emotional disorders with onset usually occurring in childhood and adolescence, unspecified mental disorder. *p = .028, **p < .001 (Chi‐squared test) [Colour figure can be viewed at wileyonlinelibrary.com]

For the detailed analysis of F‐diagnoses, focus was on the primary F‐diagnoses according to ICD‐10 at the end of hospital stay (Figure 2). In the main categories of ICD‐10, the most frequent primary diagnosis was F3 “mood [affective] disorders” (cases *n* = 796; controls *n* = 866). The most frequent subcategories were F32 “depressive episodes” (cases *n* = 319; controls *n* = 357) and F33 “recurrent depressive disorders” (cases *n* = 424; controls *n* = 449). The second most frequent primary diagnosis was F1 “mental and behavioural disorders due to psychoactive substance use” (cases *n* = 278; controls *n* = 275). These patients were mainly diagnosed with the subcategory F10 “use of alcohol” (cases *n* = 134; controls *n* = 165). These categories were equally distributed between the groups. The results showed a statistically significant difference between the groups for the primary diagnoses F2 “schizophrenia, schizotypal and delusional disorders” (cases *n* = 101; controls *n* = 181; *p* < .001) and F4 “neurotic, stress‐related and somatoform disorders” (cases *n* = 185; controls *n* = 163; *p* = .028).

**Figure 3:**
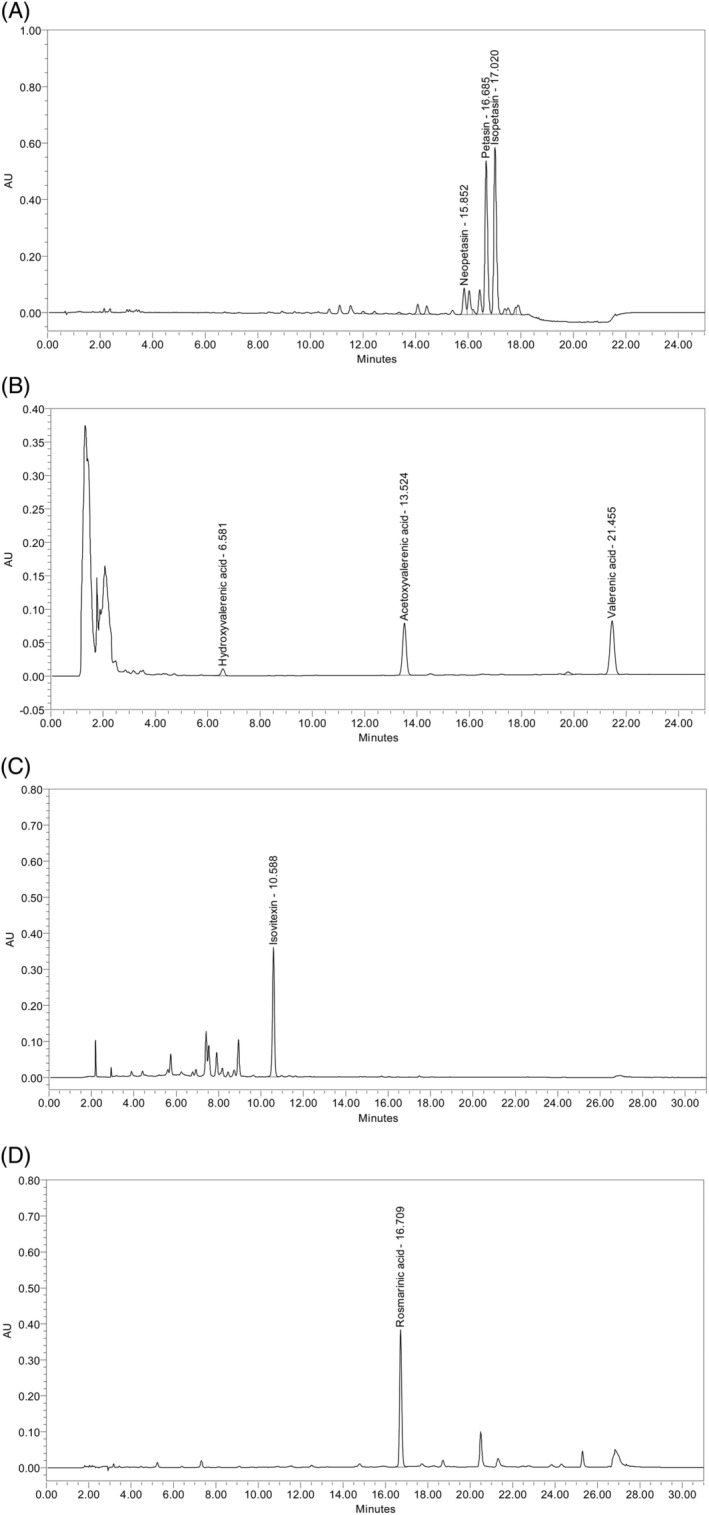
(a) High‐performance liquid chromatography (HPLC) fingerprint of *P. hybridus* dry extract performed with reversed phase HPLC (HPLC Waters H‐Class with PDA); stationary phase: C‐18 column (1.8 μm); mobile phase: gradient using acetonitrile, methanol and 0.1% solution of formic acid in water; UV‐detection at 243 nm. (b) HPLC fingerprint of valerian dry extract performed with reversed phase HPLC (HPLC 1100 Series Agilent); stationary phase: C‐18 column (5 μm); mobile phase: gradient using acetonitrile and 5 g/L solution of phosphoric acid in water; UV‐detection at 220 nm. (c) HPLC fingerprint of Passion flower dry extract performed with reversed phase HPLC (HPLC 1100 series Agilent); stationary phase: C‐18 column (3 μm) containing a C‐18 precolumn; mobile phase: gradient using 0.05 M phosphoric acid in water and acetonitrile; UV‐detection at 336 nm. (d) HPLC fingerprint of Melissa dry extract performed with reversed phase HPLC (HPLC 1100 series Agilent); stationary phase: C‐18 column (3 μm) containing a C‐18 precolumn; mobile phase: gradient using (A) 0.05 M phosphoric acid in water and acetonitrile; UV‐detection at 300 nm

### 3.2 Symptom severity and effectiveness

At baseline, controls showed a numerically comparable (6.07 ± 0.72 vs. 6.02 ± 0.67, respectively) but significantly (*p* < .022) higher CGI score (=more severe symptoms) compared with cases. No significant differences between groups were observed for the GAF score. A significant treatment effect was seen in both groups (*p* < .001, each) in both the CGI and GAF scores at the end of hospital stay. However, numerically comparable (60.0 ± 15.3 vs. 61.2 ± 15.6, respectively) but significantly (*p* = .034) lower scores (=more severe symptoms) were seen for the GAF score in controls versus cases.

To evaluate the effectiveness of the hospital stay, the CGI score, GAF score, and selected items of the AMDP system were analysed. At the end of therapy, the CGI score was similar in both groups (cases 3.36 ± 0.91, *n* = 1,421; controls 3.40 ± 0.95, *n* = 1,551; *p* = .289) and showed a significant improvement in comparison with the beginning of the therapy (*p* < .001) in both groups.

The GAF score (mean ± *SD*) was similar in both groups at the beginning of the therapy (cases 48.4 ± 12.1, *n* = 1,488; controls 47.6 ± 12.5, *n* = 1,623; *p* = .088). At the end of therapy, cases and controls were statistically different but numerically comparable (cases 61.2 ± 15.6, *n* = 1,466; controls 60.0 ± 15.3, *n* = 1,623; *p* = .034). Importantly, in both groups, there was a significant improvement from baseline to the end of therapy (*p* < .001).

At the beginning of the therapy, selected items of the AMDP system (mean ± *SD*) were partially different between the groups. The item 65 “anxious”, item 69 “restless” and item 119 “palpitations” were significantly higher for cases (anxious: 0.85 ± 0.94, *n* = 1,065, *p* = .008; restless: 0.96 ± 0.96, *n* = 1,054, *p* = .011; palpitations: 0.19 ± 0.45, *n* = 903, *p* = .032) than for controls (anxious: 0.75 ± 0.94, *n* = 1,268; restless: 0.86 ± 0.96, *n* = 1,273; palpitations: 0.14 ± 0.45, *n* = 1,056). Furthermore, cases (agitated: 0.16 ± 0.61, *n* = 1,063) were significantly less agitated (item 82) than the controls (agitated: 0.22 ± 0.61, *n* = 1,281; *p* = .020). No significant differences were found between cases and controls at the beginning of the therapy for item 83: “motor restlessness” (cases 0.32 ± 0.71, *n* = 1,063; controls 0.33 ± 0.71, *n* = 1,281; *p* = .853); item 101: “initial insomnia” (cases 1.11 ± 0.99, *n* = 936; controls 1.03 ± 0.99, *n* = 1,086; *p* = .078), item 102: “middle insomnia” (cases 1.17 ± 1.02, *n* = 934; controls 1.10 ± 1.02, *n* = 1,088; *p* = .155); item 106: “decreased appetite” (cases 0.81 ± 0.97, *n* = 929; controls 0.78 ± 0.97, *n* = 1,080; *p* = .520); item 122: “sweating” (cases 0.13 ± 0.49, *n* = 897; controls 0.15 ± 0.49, *n* = 1,052; *p* = .399); item 126: “diffuse pressure in head” (cases 0.20 ± 0.59, *n* = 905; controls 0.23 ± 0.59, *n* = 1,061; *p* = .153); and item 127: “back pain” (cases 0.25 ± 0.71, *n* = 911; controls 0.28 ± 0.71, *n* = 1,066; *p* = .278).

At the end of treatment, three items were significantly different when comparing cases and controls: item 101 “initial insomnia” (cases 0.41 ± 0.64, *n* = 824; controls 0.35 ± 0.59, *n* = 893, *p* = .046), item 102 “middle insomnia” (cases 0.40 ± 0.67, *n* = 825; controls 0.34 ± 0.60, *n* = 892, *p* = .026), and item 119 “palpitations” (cases 0.08 ± 0.29 *n* = 799; controls 0.05 ± 0.25, *n* = 878, *p* = .014). No significant differences were found between cases and controls at the end of the therapy for item 65 “anxious” (cases 0.49 ± 0.74, *n* = 935; controls 0.44 ± 0.67, *n* = 1,027, *p* = .086), item 69 “restless” (cases 0.52 ± 0.73, *n* = 933; controls 0.50 ± 0.72, *n* = 1,023; *p* = .582), item 82 “agitated” (cases 0.10 ± 0.39, *n* = 935; controls 0.11 ± 0.40, *n* = 1,028; *p* = .609), item 83 “motor restlessness” (cases 0.16 ± 0.47, *n* = 935; controls 0.17 ± 0.49, *n* = 1,029; *p* = .671), item 106 “decreased appetite” (cases 0.18 ± 0.48, *n* = 812; controls 0.21 ± 0.52, *n* = 888; *p* = .177), item 122 “sweating” (cases 0.08 ± 0.34, *n* = 801; controls 0.06 ± 0.30, *n* = 878; *p* = .275), item 126 “diffuse pressure in head” (cases 0.09 ± 0.32, *n* = 798; controls 0.10 ± 0.36, *n* = 880; *p* = .758), and item 127 “back pain” (cases 0.12 ± 0.42, *n* = 798; controls 0.16 ± 0.52, *n* = 881; *p* = .105).

Importantly, in both treatment groups, each AMDP‐item improved significantly from baseline to the end of treatment (*p* < .001).

### 3.3 Concomitant medication

Cases and controls received a plethora of concomitant medications. Therefore, the population was analysed for differences between the groups. Based on the indication of Ze 185, special focus was put on medications with psychotropic activity. The numbers of prescriptions of anxiolytics (especially benzodiazepines), hypnotics/sedatives, and antidepressants were significantly different between cases and controls (Table 2).

**Table 2:** Overview of prescribed medication in “cases” with Ze 185 and “controls” by ATC code in the drug class N, Nervous system

Drug class by ATC code	Cases Ze 185 (*n* = 1,548)	Controls (*n* = 1,704)	Total (*n* = 3,252)	*p*‐value
N02: Analgesics	571	593	1,164	.215
N02A: Opioids	60	104	164	.004
N02B: Other analgesics and antipyretics	530	533	1,063	.072
N02C: Antimigraine preparations	29	27	56	.527
N03: Antiepileptics	248	289	537	.471
N03A: Antiepileptics	248	289	537	.471
N04: Anti‐Parkinson drugs	71	97	168	.155
N04A: Anticholinergic agents	60	79	139	.284
N04B: Dopaminergic agents	11	20	31	.175
N05: Psycholeptics	1,548	1,452	3,000	<.001
N05A: Antipsychotics	900	1,035	1935	.131
N05B: Anxiolytics	655	809	1,464	.003
N05BA: Benzodiazepine derivatives	655	808	1,463	.003
N05C: Hypnotics and sedatives	940	603	1,543	<.001
N05CA: Barbiturates, plain	0	0	0	NA
N05CB: Barbiturates, combination	0	0	0	NA
N05CC: Aldehydes and derivatives	10	23	33	.046
N05CD: Benzodiazepine derivatives	9	5	14	.210
N05 CE: Piperidinedione derivatives	0	0	0	NA
N05CF: Benzodiazepine related drugs	133	174	304	.115
N05CH: Melatonin receptor agonists	0	0	0	NA
N05CM: Other hypnotics and sedatives	60	71	131	.674
N05CP: Herbal hypnotics and sedatives	0	0	0	NA
N05CX: Hypnotics and sedatives, combination excl. Barbiturates	1,548	415	1963	<.001
N05CX99: Valerian/hops extract (Ze 91019)	784	444	1,228	<.001
N05BA or N05CD benzodiazepine derivatives (total benzodiazepine)	661	809	1,470	.006
N06: Psychoanaleptics	1,160	1,150	2,310	<.001
N06A: Antidepressants	1,135	1,114	2,249	<.001
N06AA: Non‐selective monoamine reuptake inhibitors	122	131	253	.837
N06AA06: Trimipramin	63	63	126	.582
N06AB: Selective serotonin reuptake inhibitors	496	439	935	<.001
N06AF: Monoamine oxidase inhibitors, non‐selective	0	0	0	NA
N06AG: Monoamine oxidase A inhibitors	4	4	4	.892
N06AX: Other antidepressants	778	772	1,550	.005
N06AX05: Trazodone	353	320	673	.005
N06AX11: Mirtazapine	190	173	363	.055
N06AX25: Hypericum	50	29	79	.005

*Note:* Only drug classes or individual active substances for the most relevant medications with a high prevalence in patients with psychiatric disorders are depicted.

Abbreviations: ATC, anatomic‐therapeutic‐chemical; NA = not applicable.

Significantly less cases than controls received prescriptions of benzodiazepines (cases *n* = 661, controls *n* = 809; *p* = .006). The number of patients with prescribed hypnotics/sedatives was significantly higher for cases than controls (cases *n* = 940, controls *n* = 603; *p* < .001). In the group of hypnotics/sedatives, the main differences were seen for a specific valerian/hops extract (Ze 91019). Significantly more cases than controls received a prescription of this fixed extract combination (cases *n* = 784, controls *n* = 444; *p* < .001). The number of patients receiving antidepressant prescriptions was also significantly increased among cases compared with controls (cases *n* = 1,135, controls *n* = 1,114; *p* < .001). Of these, 496 cases and 439 controls received a prescription of selective serotonin reuptake inhibitors (*p* < .001). Significantly more cases than controls received prescriptions of other antidepressants (cases *n* = 778, controls *n* = 772; *p* = .005) including *Hypericum* extract (cases *n* = 50, controls *n* = 29; *p* = .005).

## 4 DISCUSSION

The number of prescriptions of benzodiazepines has considerably increased over the past decades (Agarwal & Landon, 2019; Kaufmann, Spira, Alexander, Rutkow, & Mojtabai, 2016; Verthein, Buth, Holzbach, Neumann‐Runde, & Martens, 2019). This development is giving cause for concern because of the increased risk of related adverse drug reactions posing a significant public health problem (Del Giorno, Ceschi, & Gabutti, 2017). It was concluded that understanding and addressing prescription patterns may help curb the growing use of benzodiazepines (Agarwal & Landon, 2019).

This retrospective case‐control study investigated, among other aspects, the prescriptions and use of benzodiazepines in 3,252 psychiatric patients. Our data showed that both treatment modalities for cases and controls had a comparable clinical effectiveness as indicated by CGI, GAF, and AMDP scores. Cases received significantly less prescriptions of benzodiazepine (*p* = .006) but, on the other hand, more prescriptions of hypnotics (in particular valerian/hops extract).

The demographic characteristics were similar in cases and controls. Due to the high number of patients in the case and control groups, there were small differences, which turned out to be statistically significant (e.g., for duration of hospital stay and baseline CGI score). However, these differences are probably clinically irrelevant.

In both groups, the hospital stay and the associated treatment modalities were comparably effective. This is shown by the CGI and GAF scores and selected items of the AMDP system improving significantly and comparably by the end of hospital stay. Due to the database structure of the CIS, safety relevant data could not be surveyed. Because no suspected adverse drug reactions related to the intake of Ze 185 in the period of the study were reported to the pharmacovigilance department of the marketing authorization holder of Ze 185, it may be assumed that no relevant adverse drug reactions occurred.

Most primary diagnoses were equally distributed among study subjects. However, the F2‐diagnosis (“schizophrenia, schizotypal and delusional disorders”) was statistically more prevalent in the control group. The F4‐diagnosis (“neurotic, stress‐related and somatoform disorders”) was more often diagnosed in cases. Ze 185 is not indicated in schizophrenia and associated disorders but instead for the treatment of symptoms of stress‐related and somatoform disorders. This reflects the current medicinal use of Ze 185, and thus, the data are compatible with the authorized indication. In both groups, only a limited number of patients were diagnosed. A larger number of patients would allow more definite conclusions to be drawn.

Most patients had a primary F3‐diagnosis such as F32 (“depressive episodes”) and F33 (“recurrent depressive disorders”). Depression is often associated with anxiety and somatoform disorders. This is also reflected in the study population as the second most frequent diagnosis belonged to the category F4 “neurotic, stress‐related and somatoform disorders”. Ze 185 is effective in the treatment of depression and anxiety in patients with somatoform disorders (Melzer et al., 2009). As approved by the Swiss Agency for Therapeutic Products (Swissmedic), Ze 185 is indicated for the treatment of the following complaints: nervousness, tension, restlessness, and exam anxiety, leading to symptoms such as spasmodic gastrointestinal complaints, increased irritability, occasional trouble falling asleep, and sleeping through the night. Similar symptoms such as “reduced concentration,” “disturbed sleep,” and “somatic symptoms such as agitation” are also described in the F32‐ and F33‐diagnoses. Therefore, the prescription pattern of Ze 185 reflected the authorized indication very well.

In addition to Ze 185, cases received prescriptions of other concomitant medications for the treatment of their symptoms. Focussing on psychoactive drugs influencing the nervous system, a difference in the number of prescriptions of benzodiazepines, hypnotics, and antidepressants was found. Because less patients received concomitant prescriptions of benzodiazepines among the cases compared with controls, this may suggest that Ze 185 could be a viable option to substitute benzodiazepines in patients suffering from depression and anxiety symptoms. However, to substantiate this hypothesis, a direct comparison study between benzodiazepines and Ze 185 would be necessary.

More cases than controls received prescriptions of hypnotics and antidepressants. The most frequently prescribed hypnotic was a specific valerian/hops extract. Selective serotonin reuptake inhibitors or other antidepressants, including St. John's wort, were also more often prescribed for cases. It seemed that cases being prescribed Ze 185 also had an overall higher rate of prescription for other herbal medicinal drugs. This may partially reflect the good tolerability profile and sufficient efficacy of these drugs. On the other hand, personal preferences of the patients and/or the prescribing physicians could not be excluded.

### 4.1 Strengths and limitations

Randomized, controlled clinical trials (RCT) are regarded as providing the highest evidence of proving treatment effects in evidence‐based medicine. These studies possess a high internal validity as the investigator controls for all possible confounding effects. Observational studies (cross‐sectional or case‐control studies), however, have a higher external validity, because data are observed unbiased by protocol restrictions (Carlson & Morrison, 2009). As Vandenbroucke (Vandenbroucke, 2011) pointed out in an editorial, there are four meta‐analyses contrasting RCTs and observational studies of treatments that found no large differences between the study types (Benson & Hartz, 2000; Concato, Shah, & Horwitz, 2000; Ioannidis et al., 2001; MacLehose et al., 2000).

The strength of this retrospective, case‐control study is the availability of a large sample (number of patients) and the possibility of investigating prescription patterns as it occurs in routine clinical care, therefore, providing real world evidence. The hospital has a defined public service mandate from the Canton Zurich (Zurich County, 250,000 inhabitants) and covers all levels of psychiatric diagnoses and care. Limitations are, of course, the different treatment durations and the polypharmacy of the patients. However, the polypharmacy also provided the basis for the detailed analysis of concomitant medication. Patient data from only one clinic were analysed. Characteristics of cases and controls were slightly different at baseline. Differences in the number of cases and controls between baseline and end of therapy in CGI, GAF, and AMDP items are due to missing data, which is a general limitation of retrospective studies (real world data) (Katkade, Sanders, & Zou, 2018). A multicentre study would have provided a broader picture. Therefore, with regard to effectiveness, only hypotheses on the therapeutic setting can be generated. There might be a confounding bias due to the patients' and/or physicians' preferences regarding the uses of benzodiazepines or herbal drugs that influenced the prescription pattern. Neither a causal relationship between Ze 185 and the reduction of benzodiazepine prescriptions nor a better tolerability could be inferred, due to the CIS database lacking detailed adverse event monitoring.

## 5 CONCLUSION

The broad indication of Ze 185 covers the treatment of stress‐related complaints, such as nervousness, nervous tension, agitation, and anxiousness. Patients with these symptoms are often treated with benzodiazepines. The data in the present retrospective case‐control study have certain limitations but provide some evidence that a treatment modality including Ze 185 could reduce the need for benzodiazepines. This is of general clinical interest and relevance as benzodiazepines are under ongoing discussion due to their problematic safety profile. However, to obtain a solid answer for this hypothesis, a dedicated randomized, controlled clinical trial closely monitoring drug safety is needed.

## CONFLICT OF INTEREST

MK and KS were employees of the Clienia Private Clinic Schlössli. SN, CB, CZ, and JD are/were employees of Max Zeller Söhne AG and supported the analysis of the data and the preparation of manuscript on the basis of mutual scientific agreement with MK and KS. As such, the employees confirm no bias to the publication.

## AUTHOR CONTRIBUTIONS

MK was involved in supervision, study design, data interpretation, and manuscript preparation. KS contributed to data collection and analysis. SN and CB were involved in the manuscript preparation and data interpretation. JD and CZ contributed to study design, data analysis, manuscript preparation, and revision.
